# Investigation of Stereolithography Additively Manufactured Components for Deviations in Dimensional and Geometrical Features

**DOI:** 10.3390/polym16233311

**Published:** 2024-11-27

**Authors:** Aknur Kalilayeva, Danial Zhumashev, Dongming Wei, Asma Perveen, Didier Talamona

**Affiliations:** 1 Department of Mathematics, Nazarbayev University, Qabanbay Batyr Ave. 53, Astana 010000, Kazakhstan; aknur.kalilayeva@alumni.nu.edu.kz (A.K.); dongming.wei@nu.edu.kz (D.W.); 2 Department of Mechanical and Aerospace Engineering, School of Engineering and Digital Sciences, Nazarbayev University, Astana 010000, Kazakhstan; danial.zhumashev@nu.edu.kz

**Keywords:** additive manufacturing, photogrammetry, dimensional deviation, average deviation, 3D scanning

## Abstract

The rapid investment casting (RIC) process requires a 3D-printed pattern to create a ceramic mold. Stereolithography (SLA) is a commonly used 3D printing method for pattern creation due to its ability to print complex shapes with smooth surfaces. The printing parameters can significantly affect the dimensional accuracy of the pattern. This study examines how different build orientations (0°, 45°, and 90°) affect the dimensional accuracy of parts produced using SLA. The specimens were printed using castable wax resin. They were measured to investigate the dimensional deviations using 3D scanning technology to understand the correlation between orientation and accuracy better. It was found that the orientation of the print affects the overall accuracy significantly. Parts printed at a 45° angle generally showed the smallest deviations from their nominal dimensions, except for certain features. For instance, cylindrical features showed deviations improving from −7.28% at 0° to −4.81% at 90°, while spherical features had deviations decreasing from −5.01% at 0° to −2.46% at 90°. Simple features, such as holes, exhibited minimal deviation across orientations, with the smallest error observed at 45° (1.98%). These results demonstrate different features and build orientations can affect the accuracy of the printed part differently. To ensure better accuracy, parts printed in different build orientations will require varying amounts of compensation during the design stage. By managing build orientations and controlling the inherent limitations of SLA, users can improve the print’s accuracy and meet quality standards more effectively. Research results can help industries optimize print settings and reduce dimensional errors.

## 1. Introduction

Additive manufacturing (AM) is the contemporary method of printing that slices a 3D computer-aided design (CAD) model of a part into many thin layers and builds it layer by layer. This technique accommodates a wide variety of materials suitable for AM processes, with the most common ones being polymers, metals, composites, ceramics, and glass [1]. AM technologies have revolutionized the production of complex geometries, enabling the fabrication of parts with intricate internal structures that are difficult to achieve using traditional manufacturing methods [2]. Moreover, it addresses the problem of the high amount of waste generated by conventional manufacturing processes since AM technologies require significantly less machining. Nowadays, AM is commonly used in various fields such as aerospace, automotive, biomedicine, and dentistry [3,4]. There are several sources of dimensional inaccuracies in AM technologies. The most common ones are dependent on material shrinkage, part complexity, printing parameters, and translation of CAD files to machine software [5]. Therefore, the measurement procedure needs to be conducted to assess the deviations and ensure that they are within tolerance limits. However, the inherent complexity of these geometries presents significant challenges in measurement and quality control, which are critical for ensuring the performance and reliability of AM parts in industrial applications. Measurements can be taken using two methods—contact and non-contact methods.

In the past, a contact measurement method, such as a coordinate measuring machine (CMM), was commonly accepted as a general measurement tool for different objects. The CMM is widely known for its extremely high dimensional accuracy results and ability to measure parts with different part complexity [6]. In a similar study conducted by [7], the CMM measurement results demonstrated deviations from the nominal value of less than 0.1 mm. This is consistent with the data obtained by [8]. However, in the CMM measurements taken by [9], the sphere with the nominal diameter value of 25 mm was measured and the deviation results were less than 0.1 μm. In the review paper by [10], conventional dimensional measurement tools such as Christmas Tree and H-4 systems were used. The results have shown that a maximum resolution of only 100 μms was achieved in the process.

Traditional contact measurement methods are often inadequate due to their inability to access recessed features and the slow speed of data acquisition [11]. Non-contact optical methods, including structured light scanning, have emerged as viable alternatives, offering higher speed of measurement data acquisition [12]. The non-contact measurement systems, mostly 3D laser scanners, have been developing fast in the last several decades [13]. Their comparably low cost and ability to scan complex geometries with a higher speed make their usage more common in both research and industry fields [14]. However, the measurement precision of non-contact methods is still low in comparison with contact methods [6]. A growing body of literature recognizes the importance of 3D laser scanning. In an early investigation, [15] analyzed concrete plate’s dimensional and volumetric accuracy. An average error of 2.5 mm at 200 mm nominal length and volumetric accuracy of 86.9% was achieved. The levels observed in this investigation are far below those observed by [16], which investigated the dimensional accuracy and structural performance of HLS (Hand-held Laser Scanner). The results have shown that deviations were within 0.1 mm and 95% reliability.

The change in print orientation angle can affect the overall quality of the part and production effectiveness. The parameters such as surface roughness, printing time, and hardness are greatly influenced during the process [17]. The results of [18] state that build angle and surface roughness have a monotonic relationship. A surface roughness value was rising as the angle increased. Therefore, the horizontally printed specimen showed the least surface roughness. A similar trend can be observed regarding the dependence of printing time on orientation. The part with a 0-degree inclination required the least amount of time to print, while the one at 90 degrees took the most time [19]. The influence of the build angle on hardness was investigated by the authors of [20], who claimed that a decrease in orientation resulted in improved surface hardness. The highest value for hardness was achieved at 0 degrees.

The printing built-in orientation refers to a part’s inclination angle measured from the build platform. An increase in this inclination angle alters the layer geometry, impacting the supportive forces between adjacent layers and affecting the dimensional accuracy of the object [21]. A clear relationship between dimensional accuracy and printing built-in orientation has been reported in the literature [19,22]. Among the many parameters, the printing orientation has the biggest influence on the dimensional accuracy of the part, followed by support density, model base orientation, and layer thickness [18]. The inclination of the part reportedly results in the deterioration of the staircase effect during the printing. It was found that the least amount of deviations occur when the printing orientation angle is between 45 and 60 degrees [21,23]. These results are consistent with the findings of [24], who reported that dental prosthetics printed at a 45-degree orientation with a 100 μm-layer thickness were more accurate compared to those printed at 0, 30, 60, and 90 degrees. The less accurate parts are printed in the horizontal direction, followed by a vertical orientation, which results in the highest deviations [25]. Moreover, scholarly investigations revealed that changing the build orientation from horizontal to vertical increased the average deviation by 0.01 mm [26]. Based on the result of the literature review, it can be stated that parts with a built-in orientation of 45 degrees are the most accurate. [8,18,27]. This paper investigates the dependency of dimensional deviations and geometrical features of the SLA printed pattern on the building orientation of the part. While prior studies have explored dimensional accuracy in SLA-printed parts, limited research has systematically analyzed the impact of orientation on both simple and complex geometries across a range of angles. This study fills that gap by providing a detailed analysis of dimensional deviations at 0°, 45°, and 90° orientations.

## 2. Materials and Methods

This section describes the design of the element and features, materials used, experimental and measurement procedures, and data analysis.

### 2.1. Design of the Element and Features

The test specimen used in this study was designed with a variety of geometric features to represent common challenges in the measurement of additively manufactured (AM) parts. The specimen includes a mix of cylindrical, spherical, and angular geometries and fine features that are particularly challenging to measure using traditional techniques. These features are essential for assessing the accuracy and precision of non-contact optical measurement systems, such as structured light scanning and photogrammetry.

**Figure 1:**
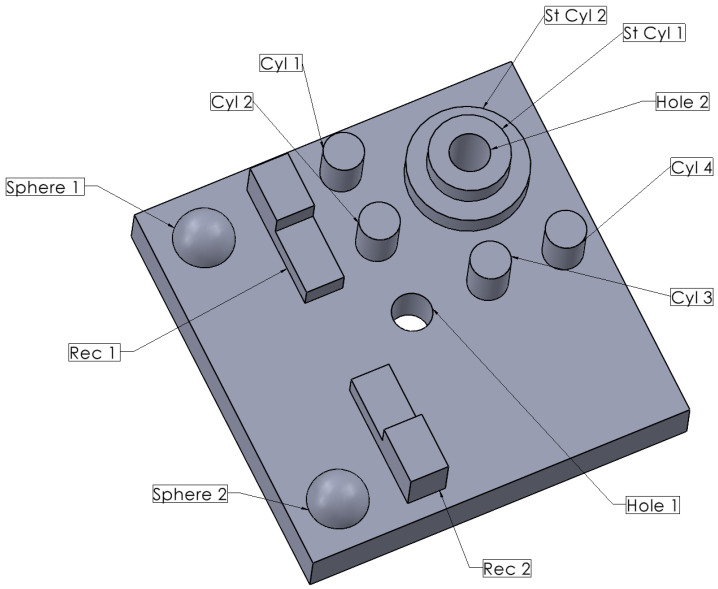
The designed test specimen with various geometric features used to evaluate the accuracy of non-contact optical measurement systems.

As illustrated in Figure 1, the specimen’s design consists of the following key features: cylindrical features (Cyl 1, Cyl 2, Cyl 3, Cyl 4) vary in diameter and height, enabling the evaluation of how well the measurement systems can capture round geometries and assess their dimensional accuracy. The specimen includes two spherical elements (Sphere 1 and Sphere 2) of different sizes. These features help in assessing the 3D scanner’s capability to accurately measure curved surfaces. Rectangle features (Rec 1, Rec 2) are designed to test the systems’ ability to capture sharp edges and flat surfaces accurately. Two through-holes of different diameters (Hole 1, Hole 2) are included to evaluate the measurement systems’ precision in capturing internal diameters and assessing the quality of circular features. Step cylindrical features (St Cyl 1, St Cyl 2) introduce additional complexity, testing the measurement systems’ ability to capture accurately stepped geometries with varying heights. According to the manufacturer’s guidelines [28,29], features are classified as “simple” or “complex” based on their shape and structure. Cylindrical and spherical features, such as D12.St-Cyl.2 and D6.Sph.1-2, are considered complex because their hollow structure or changing radii make it harder to maintain accuracy during printing, making them more prone to dimensional deviations. In contrast, simpler shapes, like holes and rectangular extensions, have consistent cross-sections, making them less resistant to inaccuracies.

**Figure 2:**
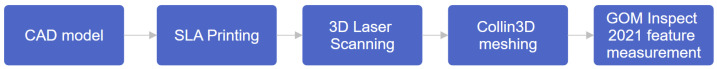
Methodology workflow.

The specimen was additively manufactured using castable wax V1 resin via SLA printer Formlabs Form 3 (Formlabs Inc., Somerville, MA, USA). The selected material was chosen due to its high accuracy and ability to print complex features. No post-processing steps were conducted beyond support removal and cleaning (Figure 2).

### 2.2. Materials

The printing was conducted with castable wax resin. This type of resin is commonly produced for dental and jewelry purposes. Castable wax resin is commonly used for SLA casting due to its high accuracy, smooth finishing, and ability to print complex features. Moreover, it contains 20 percent wax and no ash content. The properties of the resin can be seen in Table 1.

### 2.3. Experimental Procedure

**Figure 3:**
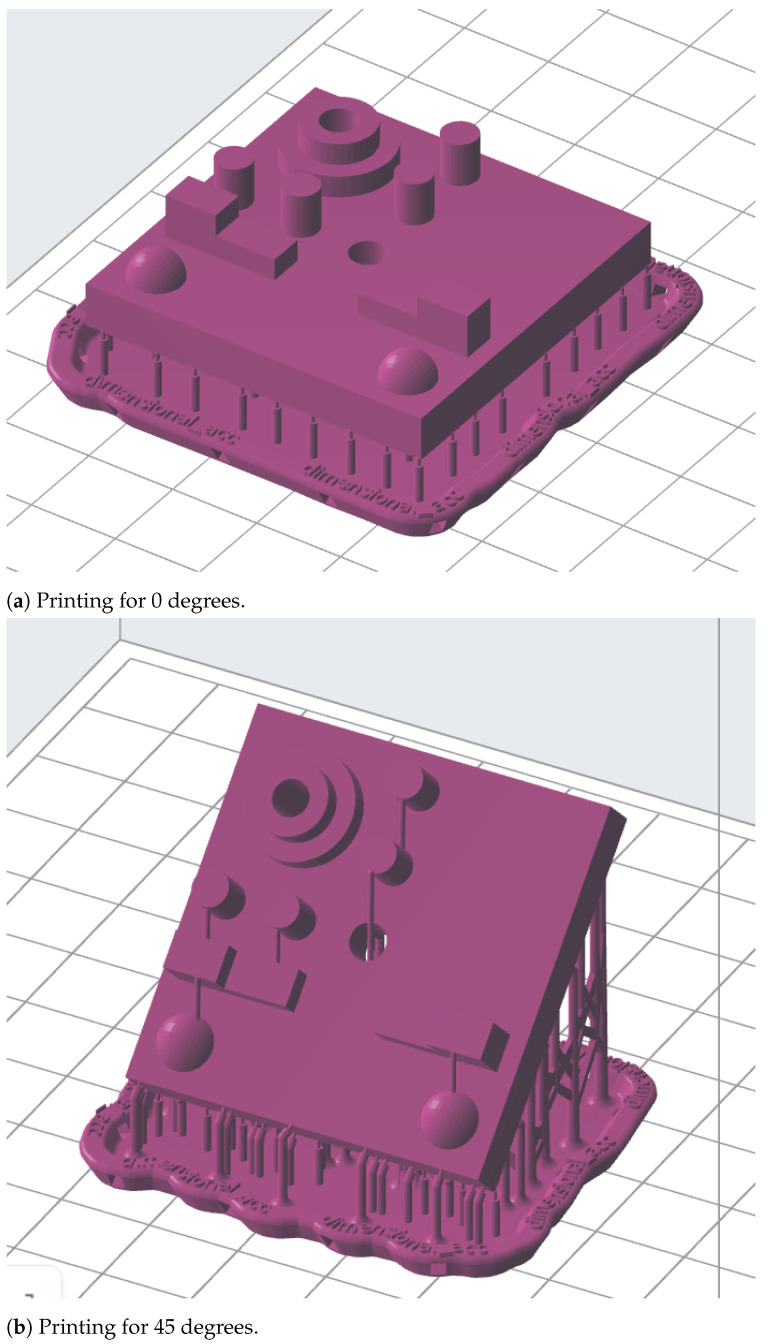

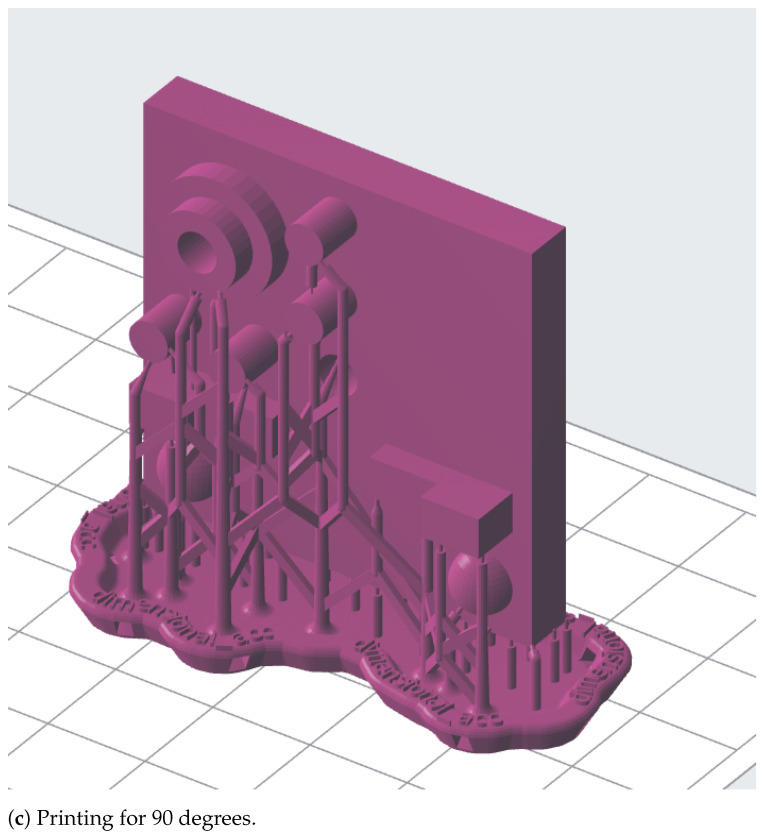
Printing at different angles: (**a**) 0 degrees, (**b**) 45 degrees, (**c**) 90 degrees.

The visual 3D model of the specimen (Figure 1) was drawn with SOLIDWORKS 2022 SP03.1 software. For SLA printing purposes, the Low Force Stereolithography (LFS) Formlabs Form 3 (Formlabs Inc., Somerville, MA, USA) printer was used with a PreForm 3.42.0 software. The parameters of the printing are shown in Table 2 and Table 3. The geometry was printed in three different built-in orientations, which are 0, 45, and 90 degrees. The visual model of the specimens with the supports can be seen in Figure 3 a–c, respectively. The process was repeated to print five samples for each orientation, resulting in a larger dataset and more accurate results. A total of 15 specimens of the measured geometry were printed. Since the ultraviolet (UV) light curing of castable wax results in part shrinkage, no post-process actions were conducted except for support removal and washing with isopropyl alcohol (IPA).

### 2.4. Measurement Systems

**Figure 4:**
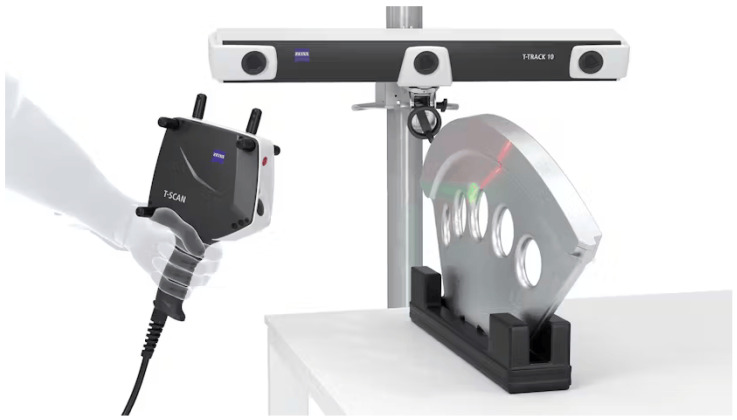
Illustration of the scanning setup [31].

The ZEISS scanning equipment setup (Figure 4) consists of three parts, as follows: the T-scan 20 laser scanner, T-track 10, and touch probe (Carl Zeiss Optotechnik GmbH, Neubeuern, Germany). However, there was no need to use a touch probe, as the equipment was calibrated prior to use.

### 2.5. Measuring Procedure

Afterward, all parts were scanned by a 3D digitization laser scanner, ZEISS T-scan 20. The calibration results of the scanner showed a standard deviation of 0.052 mm. The structured light scanner utilized a high-resolution camera in conjunction with a speckle pattern projector to capture detailed point clouds of the specimen’s surface. There were many sensors on the scanner used to capture the objects’ coordination. The scanning parameters can be seen in Table 4.

### 2.6. Data Processing and Analysis

After printing the specimens using SLA and scanning with a 3D scanner, the dimensional data (Table 5, Table 6 and Table 7) were processed to prepare them for analysis. ZEISS colin3D 9.0.0 software was used to create the triangular mesh with a mean edge length of 0.35–0.38 mm. Some small regions of the part could not be scanned, resulting in missing data. These holes were filled using the colin3D 9.0.0 function “Edit Holes”. The software closes the holes in an optimal way, i.e., by considering the neighboring area. The unnecessary data from the surroundings were selected and removed from the mesh. Next, GOM Inspect 2021 1.1.969.0 software was used to align the scanned models with their corresponding CAD designs via the “Prealignment” function. The program automatically finds the best fit between the nominal data and the mesh. Once aligned, the data were ready for statistical analysis as presented in Table 5, Table 6 and Table 7.

**Figure 5:**
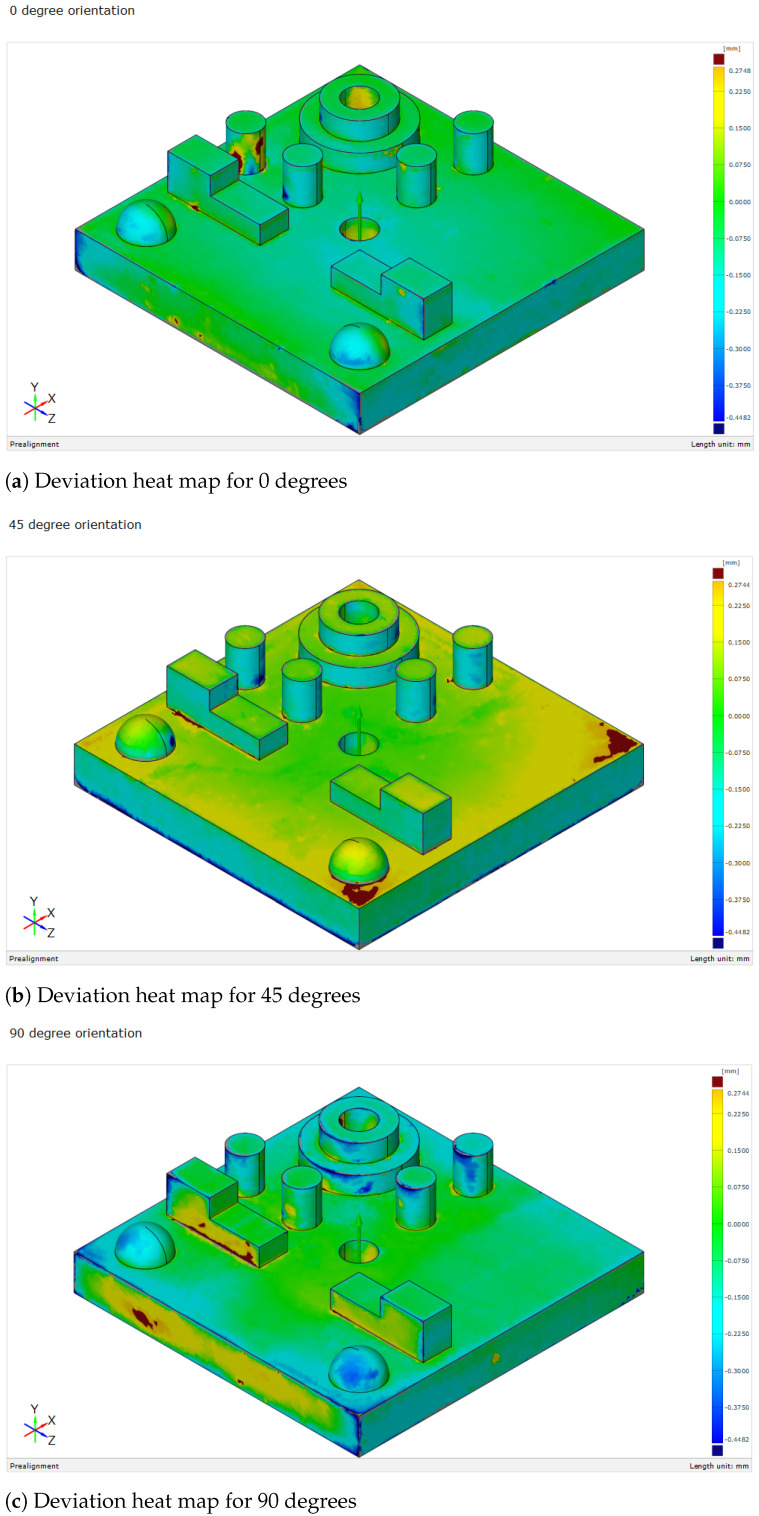
Deviation heat maps for different orientations: (**a**) 0, (**b**) 45, and (**c**) 90 degrees.

The analysis began by performing t-tests and variance assessments to determine the significance of dimensional deviations across different build orientations (0°, 45°, and 90°). Heatmaps (Figure 5a–c) highlighted the areas with the largest deviations, particularly in spherical and stepped cylindrical elements. Box plots showed that simple geometries like cylindrical holes had consistent accuracy, while more complex features had greater variability. The results indicated that complex features were prone to undersizing, likely due to resin shrinkage and inadequate support during printing. Resin shrinkage was partially accounted for by the slicing software, but complex geometries exhibited localized shrinkage that was not fully predicted, leading to under-sizing. Curing inconsistencies referred to variations in the UV curing process, where differences in exposure or resin polymerization resulted in dimensional variations, particularly in intricate or unsupported regions [32]. Key results included deviations in flat features like H2.5.Sq.1-2, which showed the smallest deviations at 45°, with −3.92%, compared to larger deviations at 0° (−4.08%) and 90° (−4.76%). Additionally, Rnd.1-2 exhibited its best accuracy at 45° with a deviation of 4.72%, improving upon the higher deviations of 4.95% at 0° and 5.96% at 90°, demonstrating the impact of orientation on dimensional accuracy.

## 3. Results and Discussion

**Figure 6:**
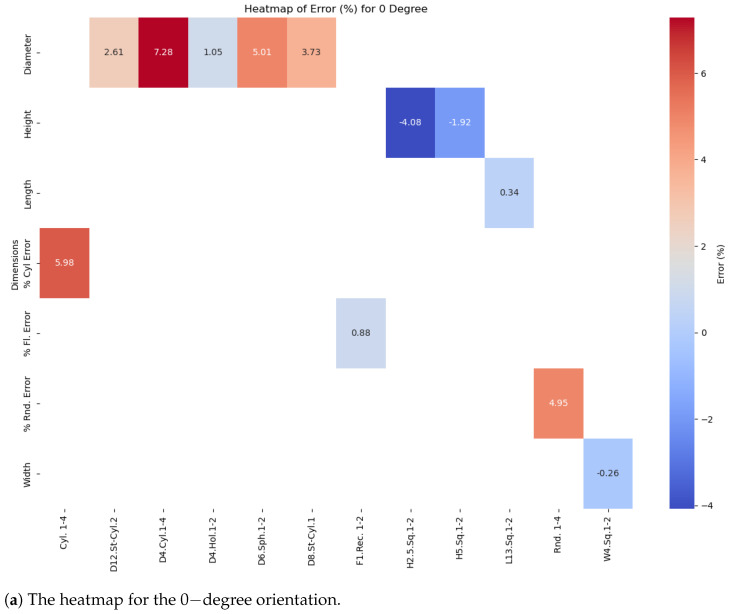

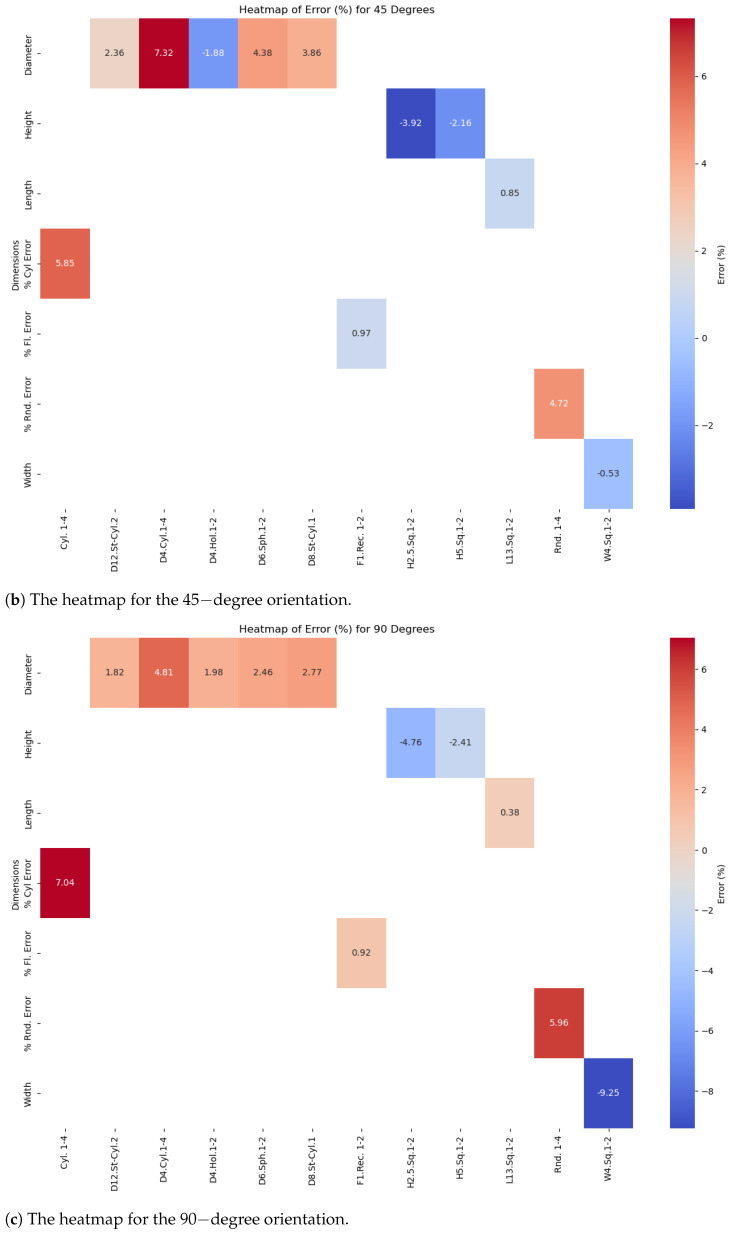
The heatmaps for different orientations: (**a**) 0 degrees, (**b**) 45 degrees, (**c**) 90 degrees.

The heatmap for the 0-degree orientation (Figure 6a) shows that while some features are close to their nominal values, others exhibit larger deviations. For instance, Diameter-D4.Cyl.1-4 has the largest positive deviation at 7.28%, followed by Cylindricity-Cyl.1-4 at 5.98% and Roundness-Rnd.1-2 at 4.95%. On the other hand, Height-H2.5.Sq.1-2 shows the largest negative deviation at −4.08%, while Diameter-D6.Sph.1-2 also has a noticeable deviation of 5.01%. At 45 degrees (Figure 6b), most features show reduced deviations, with Diameter-D12.St-Cyl.2 improving to 2.36%, and Height-H2.5.Sq.1-2 decreasing to −3.92%. However, Diameter-D4.Cyl.1-4 remains high at 7.32%. At 90 degrees (Figure 6c), some features improve, such as Diameter-D6.Sph.1-2, which decreases to 1.98%, but others, like Cylindricity-Cyl.1-4, worsen to 7.04%. Width-W4.Sq.1-2 exhibits the largest negative deviation at −9.25%. These patterns suggest that while higher orientations, like 90°, improve spherical features, 45° provides better overall dimensional accuracy for most geometries. These patterns, consistent across orientations, highlight fundamental limitations in the printing process, where geometric complexity impacts dimensional accuracy. Similar findings were reported by [7], who observed that SLA printing parameters, such as build orientation and layer thickness, significantly influence dimensional accuracy, with consistent deviations likely due to inherent challenges like resin shrinkage and curing inconsistencies during the printing process.

The heatmap for the 0-degree orientation (Figure 6a) shows the most substantial deviations among features, with the highest deviations reaching 7.28%, 5.98%, and 4.95% for Diameter-D4.Cyl.1-4, Cylindricity-Cyl.1-4, and Roundness-Rnd.1-2, respectively. These significant oversizing errors highlight challenges in maintaining dimensional accuracy for cylindrical and rounded features at this orientation. Conversely, Height-H2.5.Sq.1-2 exhibits the largest negative deviation at −4.08%, reflecting undersizing issues. This high level of deviation is likely due to limitations in the SLA printing process at 0 degrees, where minimal support for overhanging features and intricate shapes leads to increased inaccuracy.

In the 45-degree orientation (Figure 6b), deviations are generally reduced compared to the 0-degree orientation, indicating improved dimensional accuracy with better layer support. However, significant deviations still occur, particularly in Diameter-D4.Cyl.1-4 at 7.32% and Cylindricity-Cyl.1-4 at 5.85%. On the negative side, Height-H2.5.Sq.1-2 shows a deviation of −3.92%, indicating undersizing. While the 45-degree orientation improves accuracy for most features, the results highlight that complex geometries, such as cylinders and rounded edges, still face challenges despite enhanced support structures.

At the 90-degree orientation (Figure 6c), most features show smaller errors, but some complex shapes still have noticeable issues. For example, Cylindricity-Cyl.1-4 has the largest error at 7.04%, and Roundness-Rnd.1-2 is close behind at 5.96%. On the negative side, Height-H2.5.Sq.1-2 has a deviation of −4.76%, and Width-W4.Sq.1-2 shows the largest undersizing at −9.25%. While the 90-degree orientation improves accuracy for features like D6.Sph.1-2 (1.98%), more complex shapes still face challenges. Support structures at 45° and 90° orientations may play a role in mitigating some dimensional inaccuracies for complex features. These supports provide additional stability, potentially reducing deformation for intricate geometries such as D12.St-Cyl.2 and D6.Sph.1-2. However, despite these supports, the data indicates that challenges remain in achieving precise dimensional accuracy for complex shapes. This suggests that while supports contribute to improved accuracy, further optimization of support placement and density might be necessary to fully address dimensional deviations in such features.

The comparison of feature deviations across different angles, as illustrated in Figure 6a–c, demonstrates that dimensional accuracy in SLA-printed parts is strongly influenced by the build orientation, demonstrates that the accuracy of SLA-printed parts improves as the build angle increases. Complex geometries, like Cylindricity-Cyl.1-4 and Roundness-Rnd.1-2, show the largest deviations across all orientations. At 0 degrees, significant errors occur, such as 7.28% for Diameter-D4.Cyl.1-4 and 5.98% for Cylindricity-Cyl.1-4, likely due to insufficient support for intricate shapes.

At 45 degrees, the deviations become more moderate. For example, Diameter-D4.Cyl.1-4 slightly increases to 7.32%, but other features, like Height-H2.5.Sq.1-2, improve from −4.08% at 0° to −3.92%. This suggests that partial support at this angle helps mitigate inaccuracies, though complex shapes remain challenging.

By 90 degrees, deviations are further reduced. For example, Diameter-D6.Sph.1-2 improves from 5.01% at 0° to 1.98%, and Cylindricity-Cyl.1-4 increases slightly to 7.04%, but simpler shapes like Width-W4.Sq.1-2 experience the largest undersizing of −9.25%.

Overall, these findings underscore that optimizing build orientation to higher angles improves dimensional accuracy for most features, particularly for complex geometries. However, simpler shapes can still be affected by orientation changes, indicating a need for tailored support strategies to achieve consistent precision in SLA printing.

**Figure 7:**
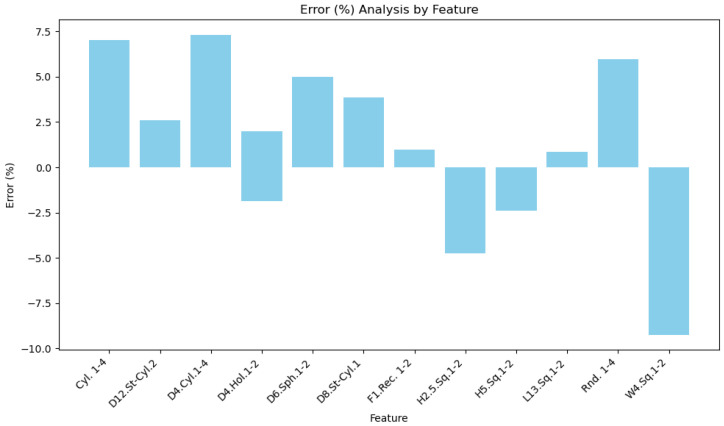
Percentage error analysis by feature.

The bar chart (Figure 7) of average percentage deviations across all features shows a wide range of errors, with the highest positive deviation observed for D4.Cyl.1-4 at approximately 7.5%, followed closely by Cyl.1-4 at around 7.0%. In contrast, the largest negative deviation is seen for W4.Sq.1-2 at nearly −9.0%, indicating significant undersizing.

Features like D6.Sph.1-2 and D12.St-Cyl.2 exhibit moderate positive deviations, reflecting some oversizing, while features such as H2.5.Sq.1-2 and H5.Sq.1-2 show consistent negative deviations, indicating slight undersizing. These results highlight the variability in dimensional accuracy depending on feature type, with complex geometries often experiencing larger deviations due to challenges in the SLA printing process. The chart shows a distribution centered around two main peaks: one near −10.0% (e.g., W4.Sq.1-2) and another around 7.5% (e.g., D4.Cyl.1-4 and Cyl.1-4). This bimodal pattern suggests the presence of two distinct deviation trends. The negative peak, caused by undersizing, corresponds to features measured consistently smaller than nominal, likely due to resin shrinkage or insufficient support during the SLA printing process. The positive peak, indicating oversizing, could result from layer accumulation or excess material deposition. Among all features, L13.Sq.1-2 shows the lowest deviation, at approximately 1.0%, indicating better dimensional accuracy for simpler shapes. Features like D12.St-Cyl.2 (approximately 2.5%) show moderate deviations, which may be attributed to their geometry, posing challenges in achieving consistent accuracy across layers and orientations. Overall, the bar chart provides insight into the error distribution across all features, highlighting how geometric complexity and orientation can impact dimensional accuracy.

The comparative analysis revealed that as the build angle increased from 0° to 90°, dimensional deviations consistently decreased, particularly for complex features like D12.St-Cyl.2. At 0°, this feature exhibited an average deviation of -2.7%, which improved to −2.3% at 45° and further to −1.9% at 90°. This demonstrates that higher build angles provide better support and reduce deformation, leading to more accurate results. However, even at 90°, significant deviations remained, indicating that adjusting the orientation alone is insufficient to resolve accuracy issues. Other factors, such as material shrinkage and layer thickness, also impact the printing process. Therefore, achieving higher precision in SLA printing requires a combination of orientation adjustments and optimization of other printing parameters.

**Figure 8:**
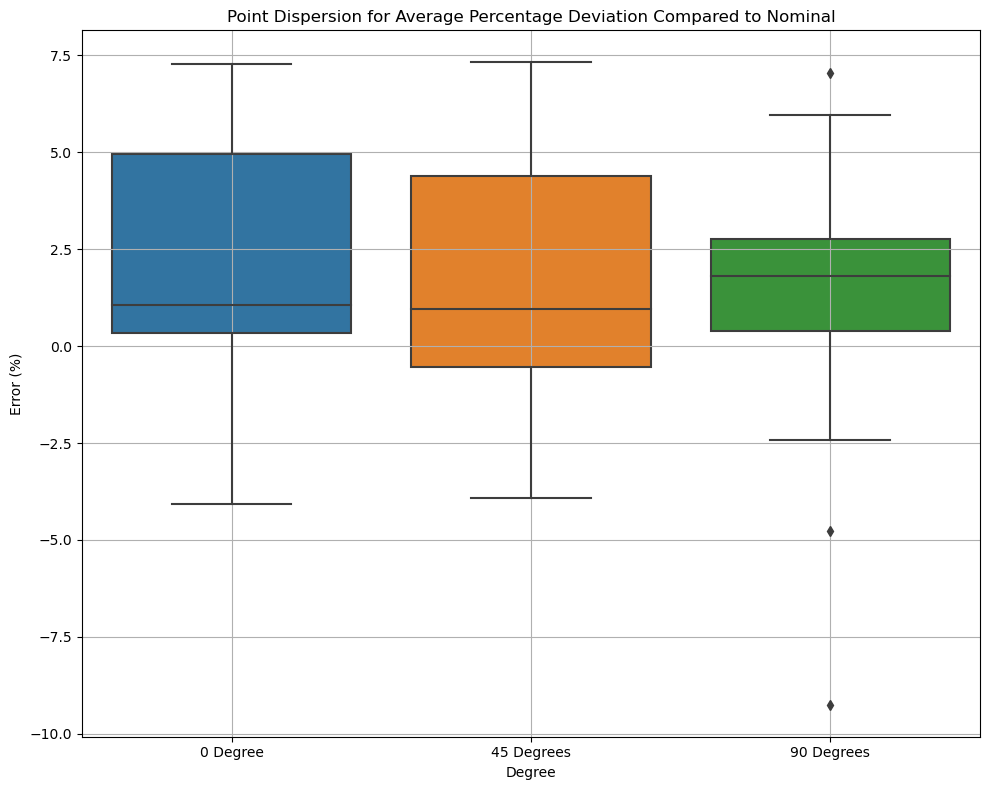
The point dispersion analysis.

Box plots in Figure 8 illustrate the distribution of percentage deviations across the three orientations (0°, 45°, and 90°). The widest interquartile range (IQR) is observed at 0°, indicating higher variability in dimensional accuracy at this orientation. At 45°, the IQR narrows, reflecting improved consistency in measurements. The smallest IQR is observed at 90°, suggesting that this orientation achieves the most uniform accuracy across features. Despite the narrowing variability, some outliers are present, particularly at 90°, which may correspond to specific features struggling with accuracy at higher angles.

Median deviations across all orientations remain close to nominal, but 0° shows a slightly higher spread compared to 45° and 90°, emphasizing the impact of orientation on accuracy. This analysis highlights that higher build angles generally improve stability and reduce variability, although outliers suggest that geometric complexity still poses challenges for certain features. The box plot analysis reveals that measurements taken at 45° are the most consistent, exhibiting the smallest variability and an average deviation of approximately −0.0313%. This suggests that the 45° orientation provides better overall stability in measurements. In contrast, measurements at 0° show slightly more variability, though they still exhibit an average negative deviation of −0.2068%, indicating good consistency. Measurements taken at 90° have the widest variability, with an average deviation of −0.227%, reflecting the greatest potential for measurement errors. Overall, the 45° orientation demonstrates the most reliable accuracy, while the 90° orientation may require additional adjustments to improve precision.

To evaluate the dimensional accuracy of the printed features, statistical t-tests were performed to compare measured values with their nominal dimensions. In Table 8, most of the features showed consistent results across angles, except for D8.St-Cyl.1 and D4.Cyl.1-4, which showed significant deviations. Namely, D8.St-Cyl.1 showed a *t*-statistic of −16.0467 (*p*-value = 0.0039), indicating a high degree of undersizing, while D4.Cyl.1-4 had a *t*-statistic of −10.0985 (*p*-value = 0.0097). Therefore, geometries with more complex shapes are more prone to errors during printing, possibly due to issues such as inadequate support structures and resin shrinkage. This proves that some geometries are more sensitive to changes during printing, resulting in less predictable dimensional accuracy.

The deviations for features such as D12.St-Cyl.2 and D6.Sph.1-2 were also statistically significant (*p* < 0.05), indicating that these errors are likely due to systematic issues rather than random variations. In addition, deviations were compared at different build angles to investigate the effect of orientation on dimensional accuracy. As a result, simpler features such as D4.Hol.1-2 and W4.Rec.1-2 showed minimal deviations, as indicated by *p*-values of 0.3965 and 0.3659 respectively, meaning that they were printed closer to their nominal dimensions. This suggests that the SLA process works more reliably with simpler geometries where alignment and support are less complex. On the other hand, complex features such as D12.St-Cyl.2, which showed a *t*-statistic of −9.8118 (*p*-value = 0.0102), and D6.Sph.1-2, which had a *t*-statistic of −6.0397 (*p*-value = 0.0263), consistently showed higher deviations. Therefore, it can be concluded that spherical and stepped cylindrical features are more prone to inaccuracies due to the limitations of the layer-by-layer manufacturing method, which can lead to underestimation, especially in areas with curved or stepped surfaces where accurate alignment and support are critical.

For features such as D12.St-Cyl.2, a statistically significant negative deviation was observed (*t*-statistic = −9.8118, *p*-value = 0.0102), which can likely be explained by shrinkage during curing and insufficient support for more complex geometries. In contrast, simpler features such as D4.Hol.1-2 did not show a significant deviation (*p*-value = 0.3965), suggesting that SLA printing is more reliable for these types of geometries. Overall, these results highlight the importance of understanding how build orientation and geometric complexity affect dimensional accuracy, as more complex shapes as D12.St-Cyl.2 require greater attention to support structures and process parameters to minimize errors. Finally, from the experimental results, it can be deduced that a sample obtained at a 45° angle is more reliable, while samples taken at a 90° position will need further consideration to minimize dispersions in measurement. The results of the one-sample t-tests on the feature averages point toward the dimensional accuracy of parts manufactured with AM processes. The feature deviations, such as those for D12.St-Cyl.2 and D6.Sph.1-2, were found to be statistically significant at p<0.05, indicating that these errors are less likely to be random and more systemic in the printing process (Table 9 and Table 10).

## 4. Conclusions

This paper investigates the dimensional accuracy of SLA-printed parts manufactured at different orientations (0°, 45°, and 90°). This research enhances existing knowledge by systematically comparing dimensional accuracy across different orientations, showing that higher angles, particularly 90°, reduce deviations in most simple geometries, while 45° consistently provides the most stable and reliable accuracy for both simple and complex features. These insights offer practical guidance for SLA printing, suggesting that orientation adjustments can significantly improve dimensional deviation, especially for simple designs. This contribution adds value to the field by offering actionable data for optimizing SLA printing in both research and industrial applications. Statistical analysis indicates that most features exhibited significant percentage deviations from their nominal dimensions, especially in complex geometries.

- Among the tested build angles, the 45° orientation consistently demonstrated the best accuracy for many features, outperforming both 0° and 90°. For example, Cyl.1-4 showed a deviation of 5.85% at 45°, improving upon the larger deviation of 5.98% at 0° and significantly better than the 7.04% at 90°. Flat features like H2.5.Sq.1-2 also showed the smallest deviations at 45° (-3.92%) compared to larger deviations at 0° (−4.08% ) and 90° (−4.76%). Additionally, Rnd.1-2 exhibited its best accuracy at 45° with a deviation of 4.72%, improving upon the higher deviations of 4.95% at 0° and 5.96% at 90°.
- Complex geometries, such as stepped cylindrical and spherical features, are particularly prone to undersizing. For instance, D12.St-Cyl.2 showed significant deviations across all orientations, with undersizing most pronounced at 0°.
- Supports at higher orientations (45° and 90°) contributed to reducing deformation in complex geometries, indicating their critical role in minimizing dimensional errors.
- Statistical tests confirmed that deviations in complex features, particularly *D12.St-Cyl.2*, were statistically significant (*t*-statistic = −9.8118, *p* = 0.0102), reinforcing the importance of orientation and support adjustments in minimizing dimensional deviations.
- The distribution of deviations shows a broad range, with significant negative deviations near −10.0% (e.g., W4.Sq.1-2) and positive deviations reaching up to 7.5% (e.g., D4.Cyl.1-4) with undersizing likely due to resin shrinkage and oversizing attributed to layer accumulation.
- Future studies should explore additional parameters, such as layer thickness and enhanced post-processing methods, to further reduce dimensional deviations and enhance the reliability of SLA-printed components.

These results confirm that spherical and stepped geometries are more prone to inaccuracies due to the layer-by-layer fabrication process, leading to greater dimensional deviations in round and curved surfaces. Statistical tests further confirmed the significance of these deviations, with D12.St-Cyl.2 displaying a *t*-statistic of −9.8118 (*p*-value = 0.0102), indicating the need for optimized processes for complex geometries. In contrast, simpler features, such as D4.Hol.1-2 and W4.Rec.1-2, showed minimal deviations from nominal values, with *p*-values of 0.8256 and 0.3659, respectively. This consistency highlights that the SLA process maintains high accuracy in simpler geometries, as fewer adjustments are needed to achieve dimensional fidelity.

In conclusion, optimizing printing parameters, particularly orientation and support structures, significantly reduces dimensional deviations in complex shapes. This should be a focus for manufacturers aiming to enhance the accuracy and reliability of SLA-printed parts. Future work should investigate the effects of layer thickness and advanced post-processing methods to further reduce deviations and improve overall part performance.

## Figures and Tables

**Table 1:** Properties of the castable wax [30].

Casting Properties	Mechanical Properties
Wax Content 20%	Young’s Modulus 220 MPa
Ash Content 0.0–0.1%	Tensile Strength 12 MPa
	Elongation Break 13%

**Table 2:** SLA printing parameters.

Parameters	Values
Layer thickness	0.050 mm
Build angle (deg)	0, 45, 90
Support density	1
Support touchpoint Size	0.60 mm
Flat spacing	6 mm
Slope multiplier	1
Raft thickness	1.5 mm
Height above raft	5 mm
Early layer merge	0.3 mm

**Table 3:** SLA printer characteristics.

Formlabs Form 3	Values
Build volume	14.5 × 14.5 × 19.3 cm
Laser spot size	85 μm
Laser power	250 mW
Optical wavelength	405 nm
XY resolution	25 μm
Operating temperature	35 Degree C

**Table 4:** Scanning parameters of T-Scan.

Scanning Parameters	Values
Exposure time	1 ms
Maximum allowed chord error	10 μm
Maximum angle of incidence	65 deg
Scan line width	100 % of 125 mm
Data rate	210,000 points/s
Line frequency	up to 330 Hz
Mean point distance	0.075 mm

**Table 5:** Dimensional deviation at 0 degrees.

Dimensions	Feature	Nominal Value	Average Measured Value	Max	Min	Average Deviation	Error (%)
Diameter	D4.Cyl.1-4	4	3.7088	3.938	3.5505	−0.2912	7.28
	D4.Hol.1-2	4	3.9578	4.083	3.7736	−0.0422	1.055
	D6.Sph.1-2	6	5.6992	5.8224	5.5504	−0.3008	5.01
	D8.St-Cyl.1	8	7.7019	7.7788	7.5954	−0.2981	3.73
	D12.St-Cyl.2	12	11.6865	11.8134	11.5895	−0.3135	2.61
Length	L13.Sq.1-2	13	12.9564	13.0609	12.771	−0.0436	0.34
Width	W4.Sq.1-2	4	4.0103	4.1668	3.9253	0.0103	−0.26
Height	H2.5.Sq.1-2	2.5	2.602	2.6593	2.538	0.102	−4.08
	H5.Sq.1-2	5	5.096	5.2166	5.0484	0.096	−1.92
Flatness	Fl.Rec. 1-2	6	0.0525	0.0899	0.0263	-	0.88
Cylindricity	Cyl. 1-4	4	0.23938	0.3514	0.1869	-	5.98
Roundness	Rnd. 1-4	4	0.19791	0.3012	0.1471	-	4.95

**Table 6:** Dimensional deviations at 45 degrees.

Dimensions	Feature	Nominal Value	Average Measured Value	Max	Min	Average Deviation	Error (%)
Diameter	D4.Cyl.1-4	4	3.7072	3.7935	3.614	−0.2928	7.32
	D4.Hol.1-2	4	4.0752	4.1773	3.9314	0.0752	−1.88
	D6.Sph.1-2	6	5.7371	5.8891	5.615	−0.2629	4.38
	D8.St-Cyl.1	8	7.6911	7.7376	7.6478	−0.3089	3.86
	D12.St-Cyl.2	12	11.7163	11.7447	11.6606	−0.2837	2.36
Length	L13.Sq.1-2	13	12.89	12.9842	12.8238	−0.11	0.85
Width	W4.Sq.1-2	4	4.0212	4.157	3.8711	0.0212	−0.53
Height	H2.5.Sq.1-2	2.5	2.598	2.6458	2.5268	0.098	−3.92
	H5.Sq.1-2	5	5.1079	5.1768	5.0554	0.1079	−2.16
Flatness	Fl.Rec. 1-2	6	0.05806	0.0783	0.0345	-	0.97
Cylindricity	Cyl. 1-4	4	0.234195	0.4261	0.1296	-	5.85
Roundness	Rnd. 1-4	4	0.18871	0.3715	0.0773	-	4.72

**Table 7:** Dimensional deviations at 90 degrees.

Dimensions	Feature	Nominal Value	Average Measured Value	Max	Min	Average Deviation	Error (%)
Diameter	D4.Cyl.1-4	4	3.8077	3.8712	3.7558	−0.1923	4.808
	D4.Hol.1-2	4	3.9208	4.0495	3.7783	−0.0792	1.98
	D6.Sph.1-2	6	5.8526	5.9714	5.7511	−0.1474	2.457
	D8.St-Cyl.1	8	7.7781	7.822	7.7101	−0.2219	2.774
	D12.St-Cyl.2	12	11.7821	11.8052	11.7427	−0.2179	1.816
Length	L13.Sq.1-2	13	12.9505	13.1568	12.7871	−0.0495	0.381
Width	W4.Sq.1-2	4	4.3699	4.48	4.2697	0.3699	−9.248
Height	H2.5.Sq.1-2	2.5	2.6191	2.7278	2.57	0.1191	−4.764
	H5.Sq.1-2	5	5.1206	5.2	5.0455	0.1206	−2.412
Flatness	Fl.Rec.1-2	6	0.05525	0.0741	0.0402	-	0.921
Cylindricity	Cyl.1-4	4	0.28151	0.3667	0.1997	-	7.038
Roundness	Rnd.1-4	4	0.23852	0.3252	0.1581	-	5.963

**Table 8:** Statistical analysis results.

Feature	*t*-Statistic	*p*-Value	Mean	95% CI
D4.Cyl.1-4	−10.0985	0.0097	−0.2553	(−0.3049, −0.2058)
D4.Hol.1-2	1.0705	0.3965	0.0352	(−0.0292, 0.0996)
D6.Sph.1-2	−6.0397	0.0263	−0.2253	(−0.2984, −0.1522)
D8.St-Cyl.1	−16.0467	0.0039	−0.2758	(−0.3094, −0.2421)
D12.St-Cyl.2	−9.8118	0.0102	−0.2737	(−0.3284, −0.2191)
L13.Rec.1-2	−2.0821	0.1728	−0.0390	(−0.0757, −0.0023)
W4.Rec.1-2	1.1597	0.3659	0.1293	(−0.0892, 0.3479)
H2.5.Rec.1-2	22.9596	0.0019	0.0816	(0.0746, 0.0885)
H5.Rec.1-2	31.8062	0.0010	0.0771	(0.0724, 0.0819)
Fl.Rec.1-2	−5.0000	0.0378	−0.0017	(−0.0023, −0.0010)
Cyl.1-2	−5.0000	0.0378	−0.0017	(−0.0023, −0.0010)
Rnd.1-2	−3.7796	0.0634	−0.0033	(−0.0051, −0.0016)

**Table 9:** Summary of dimensional deviations for SLA printed components at different orientations (part 1: dimensional deviations).

Feature	Nominal Value	0° Avg. Dev.	45° Avg. Dev.	90° Avg. Dev.
D4.Cyl.1-4	4 mm	−0.2912 mm	−0.2928 mm	−0.1923 mm
D4.Hol.1-2	4 mm	−0.0422 mm	0.0752 mm	−0.0792 mm
D6.Sph.1-2	6 mm	−0.3008 mm	−0.2629 mm	−0.1474 mm
D8.St-Cyl.1	8 mm	−0.2981 mm	−0.3089 mm	−0.2219 mm
D12.St-Cyl.2	12 mm	−0.3135 mm	−0.2837 mm	−0.2179 mm
L13.Rec.1-2	13 mm	−0.0436 mm	−0.11 mm	−0.0495 mm
W4.Rec.1-2	4 mm	0.0103 mm	0.0212 mm	0.3699 mm
H2.5.Rec.1-2	2.5 mm	0.102 mm	0.098 mm	0.1191 mm
H5.Rec.1-2	5 mm	0.096 mm	0.1079 mm	0.1206 mm
Fl.Rec.1-2	-	0.0525 mm	0.05806 mm	0.05525 mm
Cyl.1-4	-	0.23938 mm	0.234195 mm	0.28151 mm
Rnd. 1-4	4 mm	0.198 mm	0.1888 mm	0.2384 mm

**Table 10:** Summary of dimensional deviations for SLA printed components at different orientations (part 2: statistical significance).

Feature	Statistical Significance
D4.Cyl.1-4	Significant undersizing across all orientations.
D4.Hol.1-2	Minimal deviation, reliable across orientations.
D6.Sph.1-2	Significant undersizing, more accurate at 90°.
D8.St-Cyl.1	Consistent undersizing, less deviation at 90°.
D12.St-Cyl.2	Statistically significant undersizing, better at 90°.
L13.Rec.1-2	Variability observed, less significant deviations.
W4.Rec.1-2	Minimal deviations, reliable with slight oversizing.
H2.5.Rec.1-2	Consistent oversizing across orientations.
H5.Rec.1-2	Stable results, minimal oversizing across orientations.
Fl.Rec.1-2	Stable with minimal deviation.
Cyl.1-4	Consistent, slightly higher deviation at 90°.
Rnd. 1-4	Slightly higher deviation at 90°, most stable at 45°.

## Data Availability

Data are contained within the article.

## Abbreviations

The following abbreviations are used in this manuscript:

RIC	rapid investment casting
SLA	stereolithography
AM	additive manufacturing
CAD	computer-aided design
CMMs	coordinate measuring machines
HLS	hand-held laser scanner
IQR	interquartile range
CI	confidence interval
